# Longitudinal surveillance of antibiotic resistance and virulence evolution in *Clostridioides difficile*: a 4-year retrospective study of hospitalized patients in a tertiary hospital in China

**DOI:** 10.1128/spectrum.00494-26

**Published:** 2026-07-23

**Authors:** Junjie Lao, Lingli Zhang, Xinghan Huang, Guangzhi Du, Wenjie Yang, Bangxing Lin, Shenghai Wu, Hongcan Zhao, Guoqian Xiang, Liqian Wang, Xianjun Wang

**Affiliations:** 1 Department of Clinical Laboratory, Affiliated Hangzhou First People’s Hospital, School of Medicine, Westlake University, Hangzhou, Zhejiang, China; 2 The Fourth School of Clinical Medicine, Hangzhou First People’s Hospital, Zhejiang Chinese Medical University, Hangzhou, Zhejiang, China; 3 The First Affiliated Hospital, Zhejiang University School of Medicine, Liangzhu Branch, Hangzhou, Zhejiang, China; Zhejiang University School of Medicine Sir Run Run Shaw Hospital, Hangzhou, Zhejiang, China

**Keywords:** *Clostridioides difficile*, antibiotic resistance, vancomycin, metronidazole, *C. difficile *virulence genes

## Abstract

*Clostridioides difficile* (*C. difficile*) is the primary pathogen responsible for nosocomial infectious diarrhea and pseudomembranous colitis. In China, metronidazole and vancomycin are the preferred treatments for *C. difficile* infection (CDI). This study aimed to investigate the evolution of vancomycin (VA) and metronidazole (MTZ) resistance, as well as the longitudinal changes in virulence over time, using next-generation sequencing, drug susceptibility tests, and analysis of resistance and virulence genes. Additionally, we monitored the emergence of the highly virulent *C. difficile* strain RT027 and the spread and potential outbreak of *C. difficile* in the hospital setting. A random stratified sampling method was used to select 114 fecal samples from inpatients at Affiliated Hangzhou First People’s Hospital, School of Medicine, Westlake University, between 2021 and 2024. Clinical data from the enrolled patients were also collected. We conducted antigen and toxin protein detection for *C. difficile*, strain isolation and identification, drug sensitivity tests, whole genome sequencing, and bioinformatics analysis. This included comparisons of drug resistance genes, detection of toxin genes, and the construction of phylogenetic trees based on pan-genome analysis to investigate the resistance and toxin gene variations in *C. difficile*. Among the 114 samples collected from Affiliated Hangzhou First People’s Hospital, School of Medicine, Westlake University, no vancomycin- or metronidazole-resistant strains were identified. However, the average minimum inhibitory concentration (MIC) of *C. difficile* to vancomycin increased annually (*H* = 33.208, *P* < 0.05). The average MIC of *C. difficile* to metronidazole was highest in 2022 but decreased in 2023 and 2024 (*H* = 41.990, *P* < 0.05). Notably, in 2024, one *C. difficile* strain exhibited an MIC for metronidazole at the resistance threshold (2.00 μg/mL). Further Spearman correlation analysis of the strain years with drug sensitivity results revealed a positive correlation between strain years and the MIC levels of vancomycin and metronidazole (*r* = 0.528, *P* < 0.05; *r* = 0.377, *P* < 0.05). The proportion of toxin-producing strains increased annually, with 100% of strains in 2024 producing toxins, representing the highest proportion compared to the previous three years (*X*² =11.75, *P* < 0.05). Both vancomycin and metronidazole remain effective for the treatment of CDI in clinical practice. However, the sensitivity of *C. difficile* to these two drugs is gradually decreasing, and the rate of toxin gene carriage is also rising in clinical cases. No hospital outbreaks of *C. difficile* infections were identified in this study.

**IMPORTANCE:**

*Clostridioides difficile* has developed resistance to multiple antibiotics, including cephalosporins, clindamycin, and fluoroquinolones. This has exacerbated the global antibiotic resistance crisis. In China, according to current treatment guidelines, vancomycin and metronidazole are the preferred first-line drugs for treating *C. difficile* infections. However, there are reports indicating the emergence of new resistance to both vancomycin and metronidazole. Although there is extensive research on the long-term antibiotic resistance of *C. difficile* abroad, research on the continuous monitoring of antibiotic resistance and potential outbreaks of *C. difficile* in China is relatively limited. To fill this gap, we studied positive *C. difficile* strains from a tertiary general hospital in China. Through Next-Generation Sequencing (NGS), drug sensitivity testing, and analysis of drug resistance and virulence genes, we revealed the evolution of *C. difficile's* resistance to vancomycin and metronidazole, as well as changes in virulence, and monitored the spread within the hospital and potential outbreaks of *C. difficile*.

## INTRODUCTION

*Clostridioides difficile* (*C. difficile*) is a strict anaerobic, spore-forming gram-positive bacillus (1), which is the primary pathogen responsible for nosocomial infectious diarrhea and pseudomembranous colitis (2, 3). In severe cases, it can lead to life-threatening toxic megacolon (4). Its infection rate is closely related to factors such as antibiotic misuse, advanced age, and low immune function (5, 6). Among them, the RT027 strain of *C. difficile* is a highly virulent strain (7), and compared with other ribotypes, the levels of toxins A and B produced by it are 16 and 23 times higher, respectively (8). Since the early outbreak of the epidemic clone strain RT027 in 2000, research on patients with *C. difficile* infection has rapidly increased, especially in European and North American countries (9). In recent years, the RT027 clone has been sporadically detected in China, although no epidemic has occurred in China so far (10). However, with the emergence of highly virulent strains, the long-term monitoring of *C. difficile* has received greater attention.

*C. difficile* has developed resistance to a variety of antibiotics, including cephalosporins, clindamycin, and fluoroquinolones (11), contributing to the global antibiotic resistance crisis (12). Currently, fidaxomicin has not been approved for marketing by the National Medical Products Administration (NMPA) in the Chinese mainland. In China, vancomycin and metronidazole are the recommended first-line treatments for *C. difficile* infection according to the current treatment guidelines (13). However, recent reports have highlighted emerging resistance to both vancomycin and metronidazole (14). While the long-term resistance of *C. difficile* has been well-studied in foreign countries (15, 16), there has been limited research on the ongoing monitoring of resistance to these two antibiotics in China. To fill this gap, we conducted a study using random stratified sampling to select 114 *C. difficile*-positive strains from patients treated at Hangzhou First People’s Hospital between 2021 and 2024. Through next-generation sequencing, drug sensitivity testing, and analysis of resistance and virulence genes, we examined the evolution of resistance and changes in virulence to vancomycin (VA) and metronidazole (MTZ). Additionally, we monitored the emergence of the highly virulent RT027 strain, as well as hospital-acquired transmission and potential outbreaks of *C. difficile*.

## MATERIALS AND METHODS

### Fecal samples

Clinical fecal samples were collected from inpatients diagnosed with *C. difficile* infection (CDI) who were treated at Hangzhou First People’s Hospital, Affiliated to Westlake University Medical School, between January 1, 2021, and December 31, 2024. This study was reviewed and approved by the local ethics committee (KY-20231026-0246-01). Antigen detection for *C. difficile* and mass spectrometry analysis for bacterial identification were conducted, and random stratified sampling was used to select the included samples (based on the proportion of CDI-positive cases in different wards of the entire hospital). A total of 114 fecal samples positive for *C. difficile* were enrolled. The fecal samples were assigned unique numbers, and relevant clinical information, including the hospital sample identification number, gender, age, and other clinical details, was recorded from Hangzhou First People’s Hospital’s patient database.

### Detection of toxin proteins of *C. difficile*

The detection of *C. difficile* toxin proteins was performed using the *C. difficile* glutamate dehydrogenase antigen and toxin test kit (TECHLAB, USA). To prepare the mixture, 750 µL of diluent, one drop of conjugate, and 25 µL of the fecal sample were added to a test tube and mixed thoroughly. A 500 µL aliquot of the solution was transferred to the small sample well, and the test kit was incubated at room temperature for 15 min. Subsequently, 300 µL of wash buffer was added to the large reaction window and allowed to absorb completely. Two drops of the substrate were then added to the same window, and the test kit was kept at room temperature for an additional 10 min before reading the results.

### Cultivation and isolation of *C. difficile*

The culture medium used for isolation was *C. difficile* chromogenic agar medium (CDCA, produced by Qinggu Biotechnology Co., Ltd., Zhejiang Province, China). Fecal specimens were inoculated using the ethanol pretreatment method. The sample numbers of the specimens to be tested were marked on the CDCA culture medium. In an Eppendorf tube, approximately 500 µL of thawed fecal specimens were added, followed by the addition of an equal volume of 95% ethanol and mixed thoroughly. The tube was left at room temperature for 30–60 min and then centrifuged at 3,000 rpm for 10 min. The precipitate was resuspended in 300 µL of physiological saline and mixed. The two tubes of samples were then inoculated into the corresponding numbered CDCA culture media. The inoculated culture media were incubated in an anaerobic environment at 35°C ± 2°C for 48 h. In the CDCA culture medium, *C. difficile* presented as flat, rough-surfaced colonies with irregular edges and no hemolysis, forming black colonies.

### Matrix-assisted laser desorption/ionization time-of-flight mass spectrometry (MALDI-TOF MS)

The MALDI-TOF MS mass spectrometry system (Bruker, Germany) was employed for protein detection. Target and matrix coating: One microliter of the protein sample was spot-dropped onto the MSP96 target plate and allowed to dry naturally. The sample was then covered with formic acid, allowed to dry again, and subsequently covered with 1 μL of α-cyano-4-hydroxy-cinnamic acid (HCCA) matrix solution. The sample was left to dry at room temperature, allowing it to crystallize. Instrumental detection: The instrument parameters were set as follows: smartbeam laser technology (nitrogen laser, wavelength 337 nm) was used with a frequency of 60 Hz. The laser energy was adjusted to 75%–85% saturation intensity. The mass range was set from 2,000 to 20,000 Da, and detection was performed in positive ion mode. A total of 500 laser pulses were applied to each sample, and signals were automatically accumulated to a threshold of 10,000 peak intensity. Data analysis and result identification: Using Bruker Biotyper 3.1 software, the experimental spectra were compared with the MBT-CD library (which contains reference spectra for over 200 *C. difficile* strains) by calculating the similarity score (Score Value). Judging results: A score ≥2.0 indicates reliable species-level identification; a score between 1.7 and 2.0 indicates reliable genus-level identification; and a score <1.7 indicates unreliable results, requiring re-detection. In this experiment, strains with a score ≥2.0 were selected for further analysis.

### Whole genome sequencing

Whole genome sequencing (WGS) was performed using the Illumina NovaSeq 6000 platform (Illumina, USA). Reads with adapter contamination were removed, allowing for two base mismatches when compared to the adapter sequence. For paired-end sequencing, reads were discarded if the matching rate to the adapter sequence exceeded 30% for both reads or if a single read matched the adapter sequence by more than 10%. Low-quality reads were also removed; for a read to be considered low quality, if more than 30% of the read length had a base quality value (Q) < 15, it was discarded. For paired-end sequencing, if one end of a read was of low quality, both ends were removed. Genome assembly was performed using SPAdes (version 4.0.0) for bacterial genomes, which generated a contigs.fasta file containing concatenated continuous sequences. The quality of the assembly was assessed using QUAST (version 5.3.0) to verify the completeness and accuracy of the genome. Multi-locus sequence typing (MLST) was performed using the MLST script (https://github.com/tseemann/mlst) for batch MLST typing, and the genomes were submitted to the PubMLST database (https://pubmlst.org/) for MLST evolutionary clade classification. Drug resistance and toxin gene analysis was conducted by comparing the sequences with reference genes from various databases, identifying known drug resistance and toxin genes in the strains, using the ABRicate software (version 1.0.1) for multi-database comparison. The drug resistance database used was ResFinder (updated to January 14, 2025), and the toxin factor database was VFDB (updated to January 14, 2025). Genome prediction and pan-genome analysis were performed using Prokka (version 1.14.6) to predict the coding sequences (CDS) of *C. difficile*. For pan-genome analysis, Roary (version 3.12.0) was used to analyze the core and accessory genes of 117 *C. difficile* strains (including 114 experimental strains and 3 reference strains). Phylogenetic analysis was conducted using FastTree (approximate maximum likelihood method), and the tree was visualized using the online tool iTOL (https://itol.embl.de/). Core-genome Single Nucleotide Polymorphism (cgSNP) analysis was performed using Snippy (version 4.6.0), and a SNP difference matrix was generated using SNP-dist (version 0.8.2). The heatmap of the difference matrix was visualized with GraphPad Prism (version 9.5.1), and individual analysis and visualization were carried out using Cytoscape (version 3.9.1) to create a transmission network diagram.

### Drug sensitivity test for *C. difficile*

Individual colonies of *C. difficile* were selected and adjusted to a bacterial concentration of 0.5 McFarland turbidity (approximately 1 × 10⁸ CFU/mL). The bacterial suspension was then evenly spread onto the surface of Columbia blood agar using the three-layer spreading method to minimize tailing effects. E-test strips for metronidazole and vancomycin (Liofilchem, Italy) were applied by gently placing each strip onto the agar surface using sterile forceps, ensuring no air bubbles were trapped beneath the strips. One strip was applied per plate. The plates were immediately placed in an anaerobic environment and incubated at 37°C for 48 h or until the inhibition zones were clearly visible. Result interpretation: The minimum inhibitory concentration (MIC) was determined by identifying the concentration corresponding to the junction of the inhibition zone and the test strip. Quality control: For each experimental batch, ATCC 700057 was tested simultaneously to ensure accuracy (reference values for metronidazole MIC: 0.50–2.00 μg/mL and vancomycin MIC: 0.25–1.00 μg/mL). The result interpretation criteria are provided in Table 1.

**TABLE 1:** Criteria for interpretation of MIC results of antimicrobial agents for *C. difficile* (μg/mL)^*a*^

Antibacterial agents	Susceptibility (S)	Resistance (R)
VAN	≤2.00	＞2.00
MTZ	≤2.00	＞2.00

^*a*^ EUCAST 2024.

### Statistical analysis

Statistical analyses were performed using SPSS version 26.0 (IBM Corp., Armonk, NY, USA). The normality of continuous data was assessed using the Shapiro-Wilk test. Normally distributed variables (*P >* 0.05) are expressed as mean ± standard deviation ( *$\bar{x}$± s*) and were compared using independent-samples *t*-tests (for two groups) or one-way analysis of variance (ANOVA, F-test) (for multiple groups). Non-normally distributed data are presented as medians and interquartile ranges [*M* (*P*_25_*, P*_75_)] and were analyzed using the non-parametric Kruskal–Wallis H test (multi-sample rank-sum test). For correlation analysis, Spearman’s rank correlation coefficient was employed for non-parametric variables. Comparisons of categorical data (rates and proportions) were conducted using the *χ*^2^ test or Fisher’s exact test, as appropriate. For all tests, a *P <* 0.05 was considered statistically significant.

## RESULTS

### Clinical *C. difficile* isolates and patient characteristics

Among the 114 enrolled samples, 25 cases (21.93%) were positive for toxin proteins. The median age of the patients was 71 years (interquartile range [IQR], 59–86 years), which was similar to the overall median age of all patients with *C. difficile* infection in the hospital (72 years; IQR, 59–83 years). (*z* = −0.17, *P* > 0.05). Of the enrolled patients, 62 (54.39%) were male, and 52 (45.61%) were female, which closely matched the gender distribution of all *C. difficile* infection cases in the hospital (58.35% males and 41.65% females) (*χ*² = 0.611, *P* > 0.05). The distribution of enrolled patients across hospital departments showed that the four departments with the highest incidence of CDI were the Intensive Care Unit, Geriatrics, Hematology, and Oncology, which together accounted for 55.27% of the cohort. In the broader hospital population, these four departments accounted for 57.29%, indicating a highly consistent trend between the study cohort and the overall departmental distribution (*χ*² = 0.146, *P* > 0.05).

Epidemiologically, 46 cases (40.35%) were recurrent, and the median time from admission to the detection of *C. difficile* was 12.5 days (range: 4–23 days), with the median and P25 times both exceeding 72 h (3 days). Among all enrolled patients, 71 cases (62.28%) were classified as hospital-acquired CDI (HO-CDI), and 43 cases (37.72%) were classified as community-acquired CDI (CA-CDI).

Regarding prior medical history, 82 cases (71.93%) had a history of antibiotic exposure before the onset of the disease. Among these, 62 patients (54.39%) had been exposed to cephalosporins, 51 (44.74%) to carbapenems, 45 (39.47%) to penicillins, and 38 (33.33%) to fluoroquinolones. Additionally, 56 cases (49.12%) of the patients had used proton pump inhibitors (PPI). The clinical symptoms and diagnoses of the enrolled patients were categorized from mild to severe: 12 cases (10.53%) were asymptomatic, 60 cases (52.63%) had mild-to-moderate diarrhea, 33 cases (28.95%) had colitis (including pseudomembranous colitis), and 3 cases (2.63%) had severe colitis with complications (toxic megacolon and sepsis), 6 cases (5.26%) had other symptoms. Treatment outcomes showed that 48 cases (42.11%) were treated with metronidazole alone, 20 cases (17.54%) with vancomycin alone, and 17 cases (14.91%) with a combination of metronidazole and vancomycin, as detailed in Table 2.

**TABLE 2:** Sample characteristics of enrolled participants

Feature	Values
Number of positive *C. difficile* antigen (cases)	114
Age (years), *M* (*P*_25_*, P*_75_）	71 (59, 86)
Gender, Male (*n*%) (*n*%62)	62 (54.39%)
Department of patients ([cases] [%])	114 (100%)
Intensive care unit	25 (21.93%)
Gastroenterology department	25 (21.93%)
Hematology department	18 (15.80%)
Geriatrics department	13 (11.40%)
Nephrology department	10 (8.7%)
Oncology department	7 (6.14%)
Gastrointestinal and Anorectal Surgery	4 (3.51%)
General Department	3 (2.63%)
Pediatrics	2 (1.75%)
Rheumatology and Immunology Department	2 (1.75%)
Endocrinology Department	2 (1.75%)
Hepatobiliary and Pancreatic Surgery	1 (0.88%)
Cardiology Department	1 (0.88%)
Infection department	1 (0.88%)
Newly diagnosed/recurrent/unknown (cases)	63/46/5
Length of stay at admission when detected (days) *M* (*P*_25_*, P*_75_)	12.5 (4, 23)
Epidemiological classification	
Hospital-acquired CDI, (*n*%)	71(62.28%)
Community-acquired CDI, (*n*%)	43 (37.72%)
Antibiotic exposure [(cases) (%)]	82 (71.93%)
Fluoroquinolones	38 (33.33%)
Cephalosporins	62 (54.39%)
Carbapenems	51 (44.74%)
Penicillins	45 (39.47%)
Clindamycin	2 (1.75%)
Others	6 (5.26%)
PPI exposure [(cases) (%)]	56 (49.12%)
Presence of related clinical symptoms or diagnoses [(cases) (%)]	96 (84.21%)
Mild-to-moderate diarrhea	60 (52.63%)
Colitis (including pseudomembranous colitis)	33 (28.95%)
Severe colitis with complications (toxic megacolon disease, sepsis)	3 (2.63%)
Others symptoms [(cases) (%)]	6 (5.26%)
Asymptomatic [(cases) (%)]	12 (10.53%)
Drug treatment [(cases) (%)]	85 (74.56%)
Only metronidazole	48 (42.11%)
Only vancomycin	20 (17.54%)
Metronidazole and vancomycin combination	17 (14.91%)

### MLST typing and phylogenetic tree

**Fig 1:**
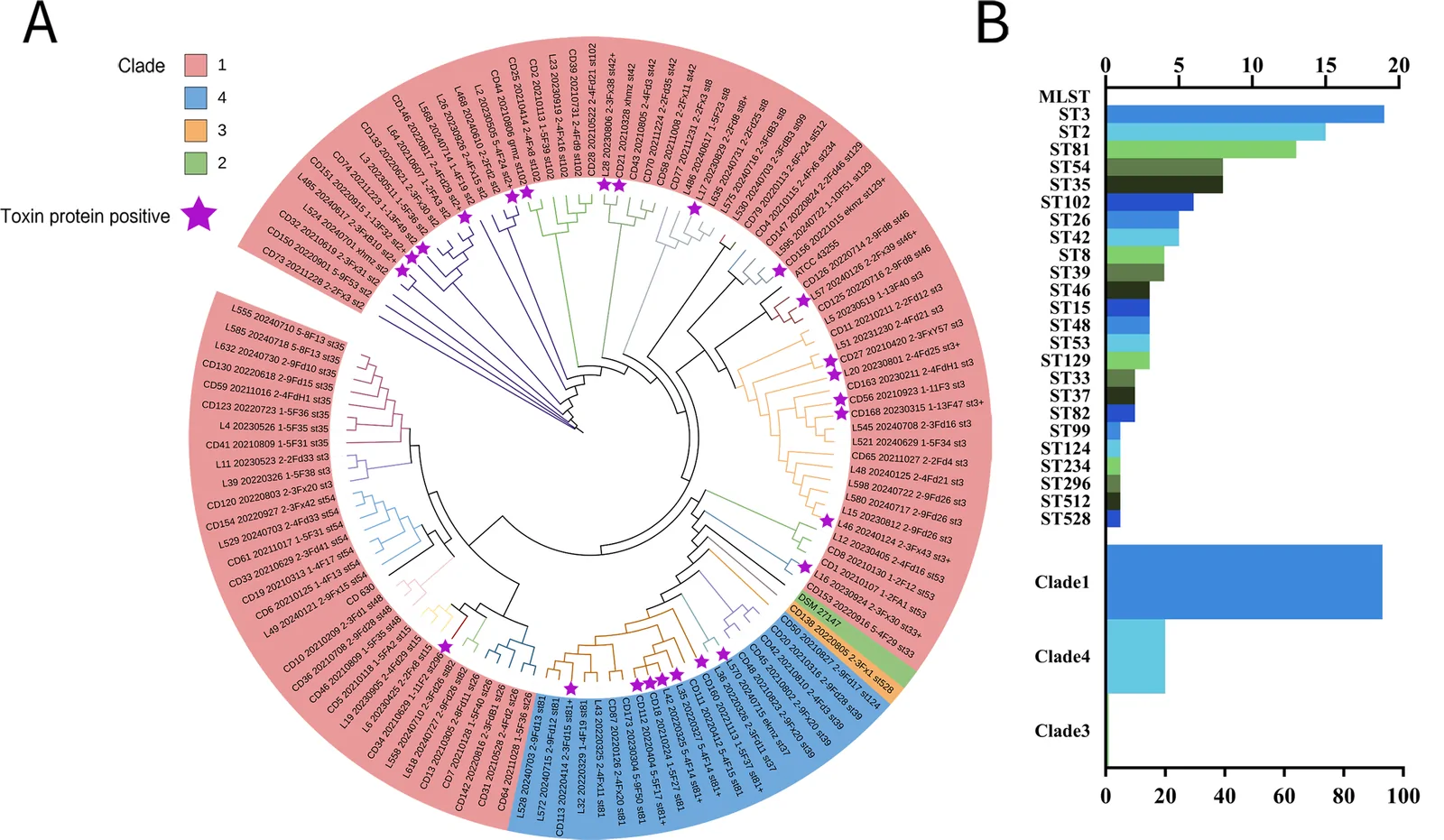
Phylogenetic tree of the genomic sequences of the enrolled samples and their typing distribution. (**A**) A phylogenetic tree was constructed based on pan-genome analysis, with four color blocks representing four evolutionary branches (Clades 1–4). Branches of the same color correspond to the same ST typing. Purple pentagrams indicate samples that tested positive for toxin proteins. (**B**) The upper part shows the number of ST types identified in the MLST typing of the enrolled samples, and the lower part illustrates the distribution and quantity of each evolutionary branch. The phylogenetic tree was constructed using the approximate maximum likelihood method in FastTree (v2.1.11), based on the core-genome alignment of 114 clinical isolates and 3 reference strains generated by Roary (v3.12.0) following annotation with Prokka (v1.14.6). Multi-locus sequence typing (MLST) was performed using the MLST script (tseemann) and validated against the PubMLST database.

To better analyze the systematic evolution of 114 *C. difficile* isolates, we comprehensively discussed the MLST typing, evolutionary branches, and toxin conditions in the evolutionary tree, and included three reference genomes (CD630, ATCC-43255, and DSM-27147) belonging to two evolutionary branches (Clades 1 and 2), totaling 117 *C. difficile* genomes. Through multi-locus sequence typing (MLST), a total of 25 distinct sequence types (STs) were identified, with each ST number representing a unique allelic profile based on seven housekeeping genes. Among them, the five most prevalent STs were ST3 (19 isolates), ST2 (15 isolates), ST81 (13 isolates), ST54 (9 isolates), and ST35 (8 isolates). The majority of the isolates belonged to Clade 1 (95 isolates, 81.20%), followed by Clade 4 (20 isolates, 17.09%) and Clade 3 (1 isolate, 0.85%) (Fig. 1B).

We used Prokka to perform gene annotation for 117 genomes (including the three reference genomes) and then used Roary for pan-genome analysis. Based on the results of the pan-genome analysis, we constructed a phylogenetic tree (maximum likelihood method) through FastTree (Fig. 1A).

### Results of drug resistance genes

**Fig 2:**
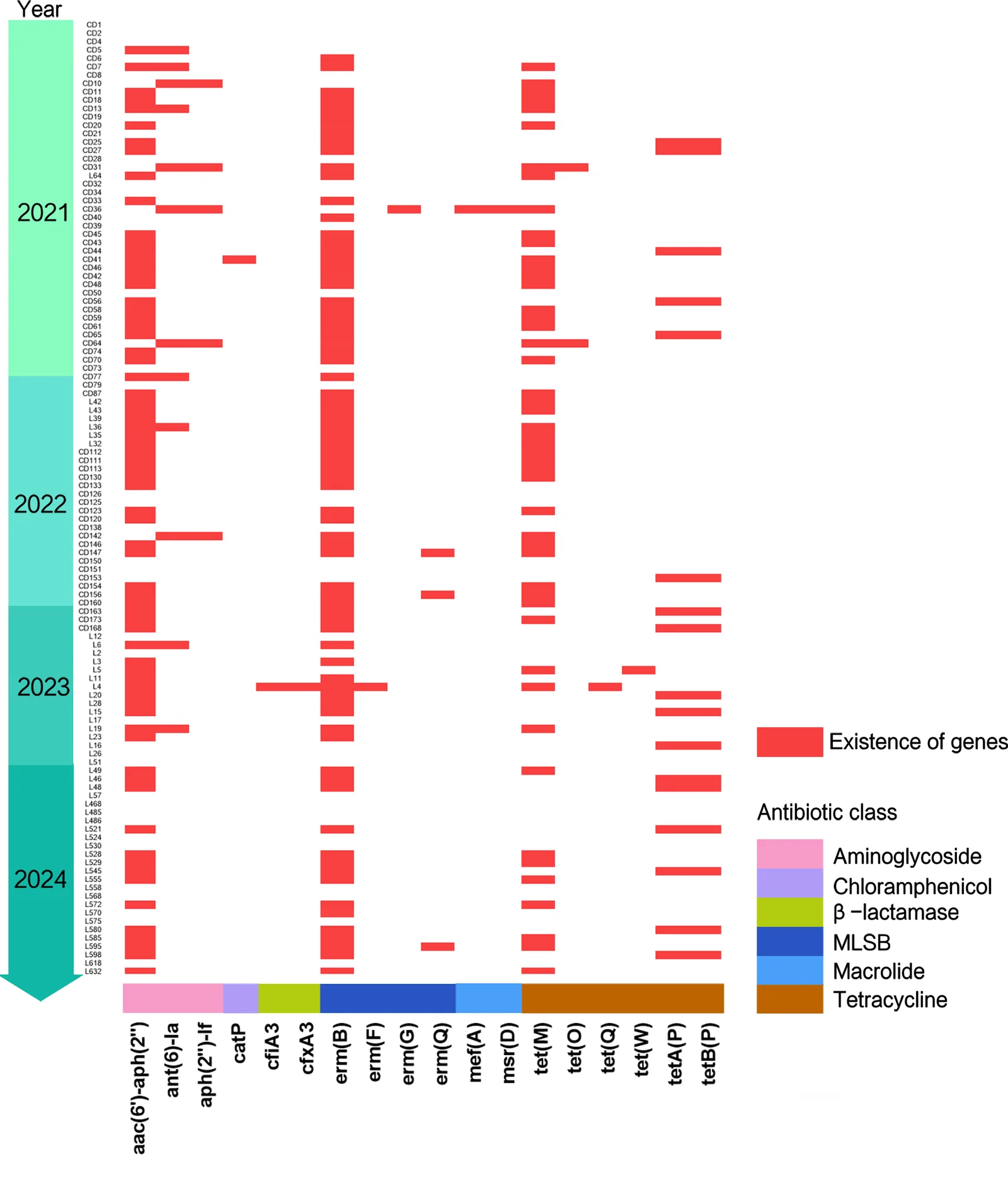
Genomic distribution of antimicrobial resistance genes (ARGs) in *C. difficile* isolates. The green arrow on the left represents the timeline. On the right, the sample ID for each item is shown. Red color indicates the presence of a specific drug resistance gene, and the names of various drug resistance genes are listed below. Different color blocks represent the various types of drugs. ARGs were identified through whole-genome sequencing analysis using the ResFinder database (updated to January 14, 2025), with a minimum identity threshold of 90% and a coverage of 60%.

We compared the genomic data of 114 *C. difficile* strains from 2021 to 2024 using ABRicate (version 1.0.1) and employed the ResFinder drug resistance database (updated to January 14, 2025). A total of 18 types of drug resistance genes were detected (Fig. 2), including *aac(6′)-aph(2″), ant(6)-Ia, aph(2″)-If, catP, cfiA3, cfxA3, erm(B), erm(F), erm(G), erm(Q), mef(A), msr(D), tet(M), tet(O), tet(Q), tet(W), tetA(P),* and *tetB(P).* However, no vancomycin-related resistance genes (*vanA, vanB,* and *VanM*) or metronidazole-related resistance genes (*nimA-nimF* and *rdxA*) were detected (Fig. 2).

From the perspective of drug resistance genes, combined with the heatmap showing the correlation between *C. difficile* drug resistance genes and time (year), the top five most abundant drug resistance genes (indicated by the vertical lines formed by dark red blocks) were as follows: *erm(B)* (77 cases), *aac(6')-aph(2'') (71 cases), tet(M)* (49 cases), *tetA(P)* (34 cases), and *tetB(P)* (34 cases).

Among the 114 strains, 84 strains (73.68%) contained at least one resistance gene. By analyzing the proportion of strains with resistance genes and the average number of resistance genes each year from 2021 to 2024, we observed that in 2021, the proportion of strains with resistance genes was 76.74%, with an average of 2.40 resistance genes per strain. In 2022, the proportion was 77.78%, and the average number was 2.33. In 2023, the proportion increased to 78.95%, with an average of 2.42 resistance genes. However, in 2024, the proportion decreased to 60.00%, with an average of 2.04 resistance genes per strain.

### Results of drug sensitivity tests

**Fig 3:**
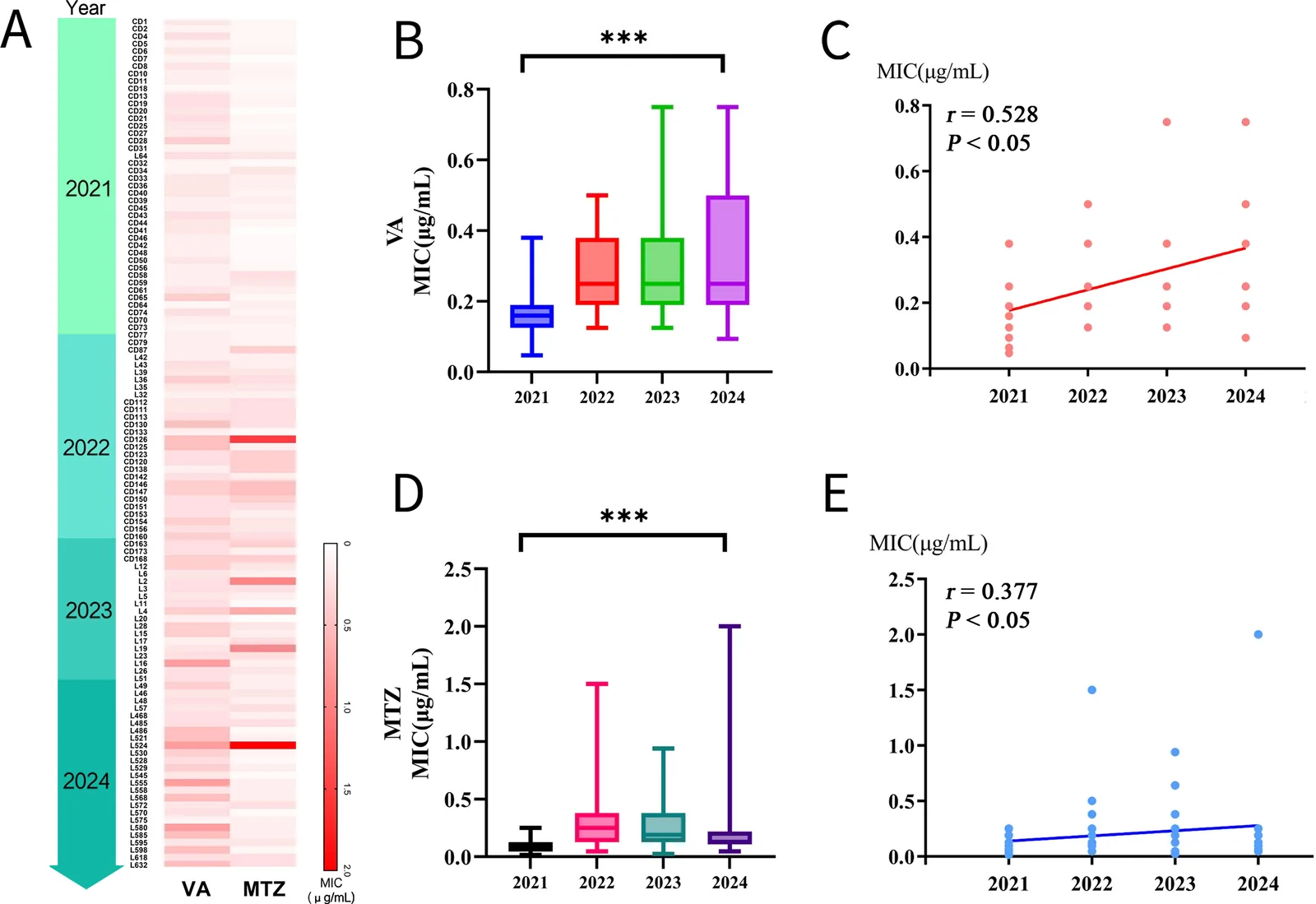
The results and analysis of vancomycin and metronidazole sensitivity tests. (**A**) The linear green color blocks represent the timeline, with the color gradually deepening from light to dark, corresponding to the period from 2021 to 2024. The right side displays the strain numbers, with samples arranged from top to bottom according to the detection time, from earliest to latest. The left red color blocks deepen from light to dark, indicating the MIC results of vancomycin and metronidazole sensitivity tests (range 0–2.0 μg/mL). (**B**) Overall results of vancomycin sensitivity tests from 2021 to 2024. (**C**) Correlation analysis diagram of vancomycin sensitivity results over time. (**D**) Overall results of metronidazole sensitivity tests from 2021 to 2024. (**E**) Correlation analysis diagram of metronidazole sensitivity results over time. Statistical analyses were performed using SPSS software (version 26.0). For comparisons involving more than two independent groups, the non-parametric Kruskal-Wallis H test (multi-sample rank-sum test) was employed. Correlation between variables was assessed using Spearman’s rank correlation coefficient. All statistical tests were two-tailed, and a *P* < 0.05 was considered statistically significant. Data visualization and figure generation were conducted using GraphPad Prism (version 9.5.1).

This study focused on monitoring the resistance of *C. difficile* to vancomycin and metronidazole. The results of the drug sensitivity tests revealed that all 114 isolated bacterial strains were sensitive to both VA and MTZ, with no resistant strains detected. To further assess the resistance trends of *C. difficile* to VA and MTZ from 2021 to 2024, we compared the MIC levels for each year’s drug sensitivity test. The comparison of the MIC levels for VA and MTZ is presented in Fig. 3B and D. Statistically significant differences were observed in the average MIC levels for both vancomycin and metronidazole across the years (VA: *H* = 33.208, *P* < 0.05; MTZ: *H* = 41.990, *P* < 0.05).

Subsequently, Spearman correlation analysis was conducted based on the strain year and drug sensitivity test results. The analysis revealed a positive correlation between the average MIC levels of vancomycin (*r* = 0.528, *P* < 0.05) and metronidazole (*r* = 0.377, *P* < 0.05) with the year of the strain, as shown in Fig. 3C and E. To present these findings more intuitively, the MIC data of the strains were visualized in a heatmap (Fig. 3A), where the red color blocks progressively deepen over time.

### Toxin phenotype and toxin gene status

**Fig 4:**
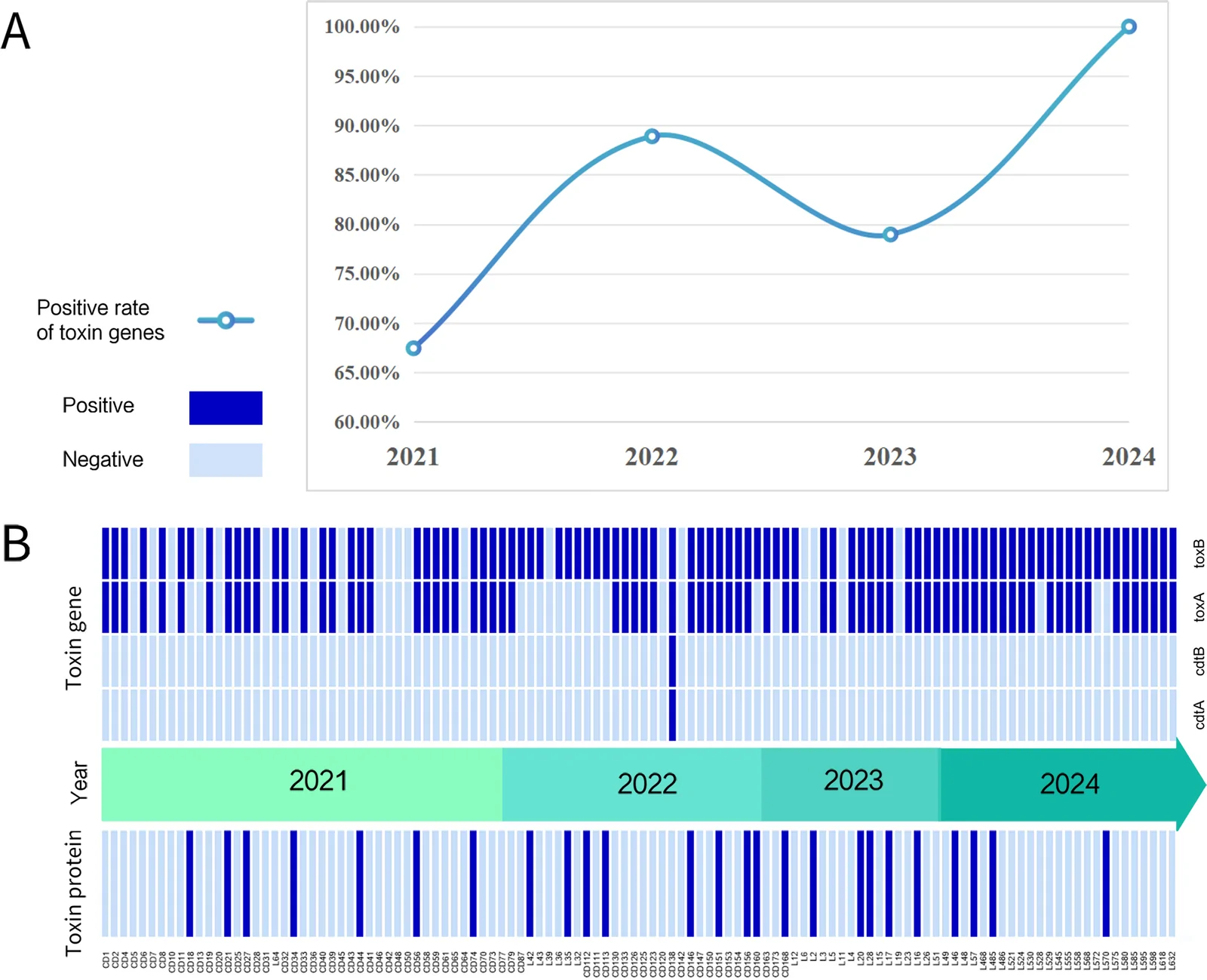
Sample toxin gene and toxin protein situation. (**A**) Trend chart of the toxin gene positive rate from 2021 to 2024. (**B**) Positive status of toxin gene and toxin protein. The linear green color blocks represent the timeline, with the color gradually deepening from light to dark, indicating the period from 2021 to 2024. Above the timeline is the positive situation of the toxin gene for each sample, and below the timeline is the positive situation of the toxin protein. Dark blue indicates a positive result, and light blue indicates a negative result. Virulence factors (VFs) were identified by aligning the assembled genomic sequences against the Virulence Factor Database (VFDB; updated to January 14, 2025) using ABRicate (version 1.0.1). The screening criteria for positive hits were defined by a minimum nucleotide identity of 90% and a minimum length coverage of 60%. The distribution of identified virulence genes across the isolates was visualized as a heatmap using GraphPad Prism (version 9.5.1).

We compared the genomic sequences of these 114 strains using ABRicate (version 1.0.1) and employed the toxin database VFDB (updated to January 14, 2025). A total of 93 samples (81.58%) were positive for toxin genes. Among these, 77 samples (67.54%) had the genotype: *cdt*(−)*toxA*(+)*toxB*(+), 15 samples (13.16%) had *cdt*(−)*toxA*(−)*toxB*(+), and one sample (0.88%) had *cdt*(+)*toxA*(+)*toxB*(+). Only 25 samples exhibited a positive toxin phenotype, accounting for 26.88% of the toxin gene-positive specimens (Fig. 4B).

The annual proportion of toxin gene-positive strains demonstrated an overall increasing trend, reaching the highest level in 2024 (100.00%) compared with the preceding 3 years. Specifically, the proportion increased from 67.44% in 2021 to 88.89% in 2022, slightly decreased to 78.95% in 2023, and subsequently rose to 100.00% in 2024 (*X*² = 11.75, *P* < 0.05) (Fig. 4A). To provide a more intuitive visualization of these findings, a heatmap illustrating the distribution of toxin genes and toxin phenotypes was generated (Fig. 4B).

### Analysis of *C. difficile* transmission based on SNP difference numbers

**Fig 5:**
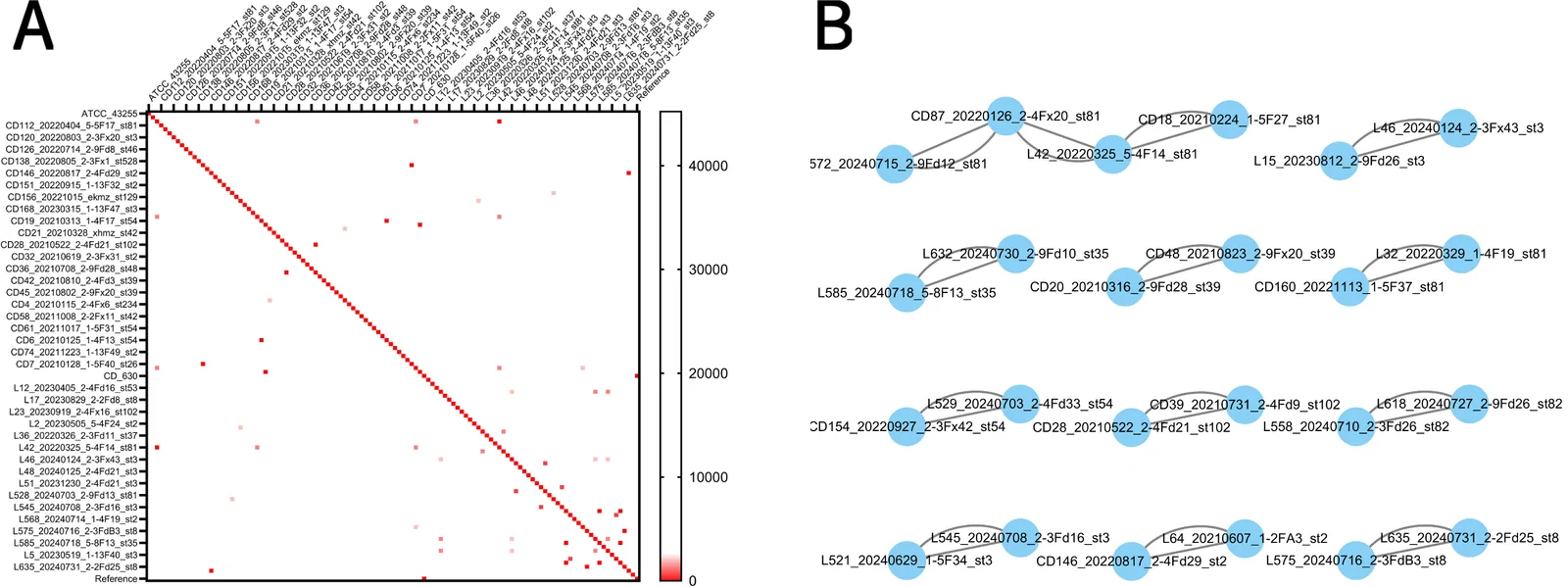
Statistical analysis of patients with SNP ≤3. (**A**) Heatmap of the SNP difference matrix (≤3). The x-axis and y-axis represent 117 genomic samples, and the scale on the right indicates the SNP difference; 0 is represented by the darkest red, and values greater than three are shown in white. Data with an SNP difference ≤3 are displayed in red in the figure, with deeper shades of red indicating smaller differences. (**B**) The two-dimensional matrix data were transformed into one-dimensional data and imported into Cytoscape to create the transmission network diagram among patients. Core-genome single-nucleotide polymorphism (cgSNP) analysis was performed using Snippy (version 4.6.0) to identify genetic variations among the *C. difficile* isolates. A SNP distance matrix was subsequently generated using SNP-dists (version 0.8.2). To visualize the genetic relatedness, a heatmap based on the SNP difference matrix was constructed using GraphPad Prism (version 9.5.1). Furthermore, the potential transmission clusters and evolutionary networks were modeled and visualized using Cytoscape (version 3.9.1).

Based on the research of Eyre et al. (17), isolates with SNP difference numbers ≤3 were considered directly genetically or transmurally related. A visual matrix heatmap was created with a cutoff value of SNP difference numbers ≤3 (Fig. 5A). The two-dimensional data from the matrix were inverted and transformed into one-dimensional data, which was then imported into Cytoscape to create the transmission network diagram. This analysis revealed 13 matrix combinations of SNP difference numbers ≤3 among different patients (Fig. 5B). However, clinical information investigations of these 13 patient groups revealed no direct evidence of patients being in the same ward or evidence of direct contact transmission.

## DISCUSSION

This study, through 4 years of clinical drug sensitivity monitoring and molecular biological analysis, found that the sensitivity of *C. difficile* to vancomycin and metronidazole is decreasing, while the carrying rate of toxin genes is steadily increasing.

The antibiotic resistance of *C. difficile* is becoming increasingly prevalent (12). The multi-drug resistance of *C. difficile* to antibiotics such as fluoroquinolones, cephalosporins, and clindamycin is worsening over time (11, 18). Notably, no vancomycin- or metronidazole-related resistance genes were found among the resistance genes, consistent with findings from some domestic studies (19). From 2021 to 2023, the proportion of strains harboring other resistant genes and the average number of resistance genes continued to increase, peaking in 2023, followed by a significant decrease in 2024. This fluctuation may be attributed to the substantial rise in the usage rates of metronidazole and vancomycin, while the antibiotic selection pressure from other drugs on *C. difficile* has decreased accordingly (20). According to the Chinese Clinical Guidelines (13), metronidazole and vancomycin are recommended as the preferred treatments for patients with CDI, while fidaxomicin has not yet been approved for use by the National Medical Products Administration of China (NMPA). Therefore, this study focused on the resistance situations of these two widely used antibiotics. The drug sensitivity results indicated that no resistant strains to vancomycin and metronidazole were found among the enrolled samples, which is consistent with the findings of Xin Wen et al.’s study (21), suggesting that these two antibiotics still have significant therapeutic effects on CDI in this hospital. A statistical analysis of the MIC levels of vancomycin and metronidazole against *C. difficile* from 2021 to 2024 revealed that the average MIC level of vancomycin increased each year. For metronidazole, the average MIC was highest in 2022 but decreased in 2023 and 2024. However, in 2024, a *C. difficile* strain was found with an MIC of 2.00 μg/mL, which is at the resistance breakpoint (2.00 μg/mL). We analyzed the changing trends of MIC50 and MIC90 for the two drugs over a 4-year period. The results showed that the changing trends of MIC90 and the average MIC values were highly consistent over the 4-year period (Table S1). Spearman correlation analysis, combining the strain year and the drug sensitivity test results, showed a positive correlation between the year of the strain and the MIC levels of both vancomycin and metronidazole. These findings suggest that over time, the sensitivity of *C. difficile* to vancomycin and metronidazole is continuing to decline, a trend that is consistent with a recent survey conducted in a pediatric tertiary hospital in Shanghai (14). In that study, resistance rates for both metronidazole and vancomycin significantly exceeded those previously reported in China (21, 22) and other regions abroad (18, 23). In our study, although MIC levels have increased, they have not yet crossed the clinical resistance threshold. Therefore, they do not directly lead to clinical failure of vancomycin or metronidazole treatment at present. However, this suggests that we need continuous genomic monitoring to prevent any potential qualitative changes in the future. It should be noted here that compared with the agar dilution method recommended by CLSI, the use of the E-test may have limitations. However, previous studies (24, 25) have shown that these two methods have a high degree of consistency in the classification of drug resistance. The E-test is particularly suitable for clinical monitoring because it has strong operability in retrospective clinical studies, and we have added quality control in each experiment to ensure the stability of the experiment.

This study detected the toxin genes of *C. difficile* strains and observed a continuous increase in the proportion of strains carrying toxin genes from 2021 to 2024. This suggests a general trend toward the development of toxin-producing *C. difficile* strains. The proportion of strains with the toxin gene was 81.58%, which is consistent with the findings of Deiziane V. S. Costa et al. (26) (80.4%). Although we found that up to 81.58% of the strains carried the toxin gene, only 26.88% actually showed positive for the toxin protein. This difference between the gene and the phenotype is reasonable. First, toxin expression is strictly regulated by amino acid levels, carbon metabolism, etc. Having the gene does not mean that expression is activated. Second, in this study, the *C. difficile* glutamate dehydrogenase antigen and toxin detection kit (USA, TECHLAB) indicates that the detection level of toxin A in this kit is ≥0.63 ng/mL, the detection level of toxin B is ≥0.16 ng/mL, and the detection level of glutamate dehydrogenase is ≥0.8 ng/mL. Given the sensitivity threshold limitations of immunological detection methods, some strains with lower toxin expression levels may not be detectable. Additionally, the study by Costa et al. (26) found that the antibiotic sensitivity of *C. difficile* strains with positive binary toxin genes tended to decrease. In this experiment, the only binary toxin-positive sample did not exhibit significantly higher MIC values for VAN and MTZ compared to the other negative samples. However, the MIC values for both drugs and the positive rates of toxin genes A and B all showed a synchronous increase over the years. This trend offers valuable insights for future research on the correlation between drug resistance and toxin gene presence.

Statistical analysis of clinical information on *C. difficile* infection revealed that the departments with the highest number of patients were the intensive care unit, oncology, hematology, and geriatrics, collectively accounting for 55.27%. Patients in these departments often experience intensive use of broad-spectrum antibiotics (27), have prolonged use of proton pump inhibitors (28), and are typically elderly (29) or in an immunosuppressed state (30), frequently accompanied by underlying diseases (31). These factors, combined with the need for long-term or repeated hospitalization, significantly increase the risk of *C. difficile* infection. The recurrence rate of *C. difficile* infection in this study cohort was 40.35%, consistent with the findings of Christine Leong and Sheryl Zelenitsky (32), where 20%–30% of CDI patients experienced their first recurrence, and 40%–60% of the cases experienced a second or more recurrences. The proportion of healthcare-associated infections (HAIs) related to CDI was 62.28%, emphasizing that the medical environment remains the primary site for *C. difficile* transmission. Since the subjects of this cohort study were hospitalized patients, the proportion of CDI in healthcare settings was slightly higher than the findings of Alice Y. Guh et al. (33), where healthcare-associated CDI accounted for 51.39%–65.82% of the cases.

In investigating the medication history of CDI patients prior to infection, it was found that the use of broad-spectrum antibiotics such as cephalosporins, carbapenems, penicillins, and fluoroquinolones significantly increased the risk of *C. difficile* infection (CDI) due to their disruptive effects on the microbial flora (34). A total of 63 cases (55.26%) had been exposed to two or more antibacterial drugs, and more than half of the patients had a history of short-term multi-drug combination therapy. The rate of PPI use was high (49.12%), which aligns with the meta-analysis by Matilda Finke et al. (35), indicating that with increased dosage and duration of PPI treatment (36, 37), the risk of CDI may rise. PPIs weaken the acidic environment of the gastrointestinal tract, inhibiting the growth of normal intestinal bacteria, such as lactobacilli and bifidobacteria, while promoting the germination of *C. difficile* spores and toxin expression. This leads to an increase in the proportion of secondary bile salts in the intestine, activates the germination signaling pathway of *C. difficile* spores, and upregulates the expression of toxin genes (*tcdA/tcdB*) (38, 39). In the MLST typing, the analysis of 25 ST types revealed a high degree of genetic diversity in the transmission of *C. difficile*. The most frequent ST types were ST3, ST2, and ST81, which is consistent with previous domestic research and meta-analyses (14, 40), suggesting that these dominant typing patterns may have a stronger ability to survive and transmit in hospital-acquired infections. The distribution of evolutionary branches was primarily Clade 1, followed by Clade 4, which also aligns with previous domestic research results (19). Furthermore, through phylogenetic tree and SNP difference number analysis, no outbreak of hospital-acquired infection was identified in this study. It is possible that other intermediate transmission routes in hospital-acquired transmission, such as contamination of medical facilities or medical staff acting as intermediaries, may have been overlooked as potential hosts or transmission links.

In conclusion, our results indicate that the sensitivity of *C. difficile* to metronidazole and vancomycin is progressively decreasing among hospitalized patients, while the proportion of strains carrying toxin genes is increasing. This highlights the need for enhanced monitoring of both drug resistance and virulence in clinical practice (24, 25). To address the growing challenges posed by *C. difficile* drug resistance and virulence, it is crucial to explore more targeted treatment strategies, such as switching to fidaxomicin (41) or considering fecal microbiota transplantation (42). These approaches may help mitigate the increasing risks associated with *C. difficile* infections in China.

## Supplementary Material

Reviewer comments

## Data Availability

The raw sequencing data generated in this study have been deposited in the NCBI Sequence Read Archive (SRA) database under BioProject accession number PRJNA1456209. All other associated metadata are available in the supplemental material.

## SUPPLEMENTAL MATERIAL

Table S1 spectrum.00494-26-s0001.xlsx Drug sensitivity results of *Clostridioides difficile* for vancomycin (VA) and metronidazole (MTZ) from 2021 to 2024.

OPEN PEER REVIEW reviewer-comments.pdf An accounting of the reviewer comments and feedback.

ASM does not own the copyrights to Supplemental Material that may be linked to, or accessed through, an article. The authors have granted ASM a non-exclusive, world-wide license to publish the Supplemental Material files. Please contact the corresponding author directly for reuse.
