# Supplementary material for: Longitudinal surveillance of antibiotic resistance and virulence evolution in Clostridioides difficile: a 4-year retrospective study of hospitalized patients in a tertiary hospital in China
Source: Microbiol Spectr. 2026 Jul 23;14(9):e00494-26. doi: 10.1128/spectrum.00494-26 (PMC13532177; doi:10.1128/spectrum.00494-26)
Supplement: Supplementary file 1 [file reviewer-comments.pdf]

AMERICAN SOCIETY FOR MICROBIOLOGY | Microbiology Spectrum

Longitudinal Surveillance of Antibiotic Resistance and Virulence Evolution in *Clostridioides difficile*: A Four-Year Retrospective Study of Hospitalized Patients in a Tertiary Hospital in China

Junjie Lao, Lingli Zhang, Xinghan Huang, Guangzhi Du, Wenjie Yang, Bangxing Lin, Shenghai Wu, Hongcan Zhao, Guoqian Xiang, Liqian Liqian Wang, and Xianjun Wang

Corresponding Author(s): Xianjun Wang, Hangzhou First People's Hospital

Review Timeline:

Submission Date:	February 10, 2026
Editorial Decision:	March 24, 2026
Revision Received:	June 1, 2026
Accepted:	June 29, 2026

Editor: Zhi Ruan

Reviewer(s): The reviewers have opted to remain anonymous.

Transaction Report:

(Note: With the exception of the correction of typographical or spelling errors that could be a source of ambiguity, letters and reports are not edited. The original formatting of letters and referee reports may not be reflected in this compilation.)

DOI: https://doi.org/10.1128/spectrum.00494-26

Re: Spectrum00494-26 (**Longitudinal Surveillance of Antibiotic Resistance and Virulence Evolution in *Clostridioides difficile*: A Four-Year Retrospective Study of Hospitalized Patients in a Tertiary Hospital in China**)

Dear Dr. Xianjun Wang:

Thank you for the privilege of reviewing your work. Below you will find my comments, instructions from the Spectrum editorial office, and the reviewer comments.

Please return the manuscript within 60 days; if you cannot complete the modification within this time period, please contact me. If you do not wish to modify the manuscript and prefer to submit it to another journal, notify me immediately so that the manuscript may be formally withdrawn from consideration by Spectrum.

Revision Guidelines
To submit your modified manuscript, log into the submission site at https://spectrum.msubmit.net/cgi-bin/main.plex. Go to Author Tasks and click the appropriate manuscript title to begin. The information you entered when you first submitted the paper will be displayed; update this as necessary. Note the following requirements:

• Upload point-by-point responses to the issues raised by the reviewers in a file named "Response to Reviewers," NOT in your cover letter.
• Upload a compare copy of the manuscript (without figures) as a "Marked-Up Manuscript" file.
• Upload a clean .DOC/.DOCX version of the revised manuscript and remove the previous version.
• Each figure must be uploaded as a separate, editable, high-resolution file (TIFF or EPS preferred), and any multipanel figures must be assembled into one file.
• Any supplemental material intended for posting by ASM should be uploaded with their legends separate from the main manuscript. You can combine all supplemental material into one file (preferred) or split it into a maximum of 10 files with all associated legends included.

For complete guidelines on revision requirements, see our Submission and Review Process webpage. Submission of a paper that does not conform to guidelines may delay acceptance of your manuscript.

Data availability: ASM policy requires that data be available to the public upon online posting of the article, so please verify all links to sequence records, if present, and make sure that each number retrieves the full record of the data. If a new accession number is not linked or a link is broken, provide Spectrum production staff with the correct URL for the record. If the accession numbers for new data are not publicly accessible before the expected online posting of the article, publication may be delayed; please contact production staff (Spectrum@asmusa.org) immediately with the expected release date.

Publication Fees: For information on publication fees and which article types are subject to charges, visit our website. If your manuscript is accepted for publication and any fees apply, you will be contacted separately about payment during the production process; please follow the instructions in that e-mail. Arrangements for payment must be made before your article is published.

ASM Membership: Corresponding authors may join or renew ASM membership to obtain discounts on publication fees. Need to upgrade your membership level? Please contact Customer Service at Service@asmusa.org.

The ASM Journals program strives for constant improvement in our submission and publication process. Please tell us how we can improve your experience by taking this quick Author Survey.

Thank you for submitting your paper to Spectrum.

Sincerely,

Professor Zhi Ruan
Editor
Microbiology Spectrum

Reviewer #1 (Comments for the Author):

This study provides useful surveillance data on C. difficile isolates from a single hospital in China; however, the interpretation of the findings appears somewhat overstated. Although minor increases in MIC values and toxin gene prevalence were observed, the data do not yet demonstrate a true rise in antimicrobial resistance or virulence. The study also does not discuss the role of fidaxomicin, which is recommended as a first-line treatment for C. difficile infection, nor does it clarify that metronidazole and vancomycin are generally considered alternative or adjunct therapies. Additionally, the authors should consistently use C.

difficile and minimize the use of the full name to maintain clarity and adherence to standard nomenclature. Further research with larger sample sizes, multicenter datasets, and longer monitoring periods is needed to confirm the trends suggested and to clarify the implications for current treatment strategies.
Summary Critique:
The study concludes that C. difficile resistance to vancomycin and metronidazole is gradually increasing, but the data do not fully support this claim. According to the results, all 114 isolates remained susceptible to both antibiotics based on EUCAST criteria, and no resistance-associated genes (such as vanA, vanB, or nim genes) were detected. Although the authors report an increase in MIC values over time, these values remain within the susceptible range, meaning there is no confirmed clinical resistance. Therefore, the evidence only indicates minor MIC variation, not true resistance development.

The interpretation that increasing MIC values represent emerging resistance may be misleading. MIC values naturally fluctuate due to several factors, including laboratory conditions. The authors should stick with reference method for testing the MIC for anaerobes and compare it with E-test (commercial). Because the reported MIC values remain below the resistance breakpoint, the observed trend may not represent a biologically meaningful change. How about MIC50 and MIC90
The study includes 114 isolates collected over four years, averaging fewer than 30 strains per year. This relatively small sample size limits the ability to detect reliable resistance trends. Furthermore, the research was conducted in a single hospital, which restricts the generalizability of the findings.
The authors also argue that C. difficile virulence is increasing, based on the rising proportion of strains carrying toxin genes. However, the results show a significant discrepancy between toxin gene detection (81.58%) and toxin protein expression (26.88%). This difference suggests that the presence of toxin genes does not necessarily translate into toxin production. Several biological factors could explain this discrepancy, that not explored in this MS.
The study utilizes WGS and resistance gene analysis, which is a strength. However, the analysis does not fully integrate genomic findings with phenotypic antibiotic susceptibility or clinical data.
The study covers a four-year period with very limited sample size, which is relatively short for assessing long-term antimicrobial resistance trends.

Reviewer #3 (Comments for the Author):

This study investigates longitudinal trends in antimicrobial susceptibility and virulence characteristics of Clostridioides difficile isolates collected between 2021 and 2024 at a single tertiary hospital in China. Using drug susceptibility testing, whole-genome sequencing, and bioinformatic analyses, the authors report that no fully resistant strains to vancomycin or metronidazole were identified. However, minimum inhibitory concentrations (MICs), particularly for vancomycin, increased over time, and one isolate in 2024 reached the metronidazole resistance threshold. The study also documents a rising proportion of toxin-producing strains, with all isolates in 2024 carrying toxin genes. Overall, the manuscript addresses an important clinical topic and incorporates genomic approaches to examine resistance and virulence evolution. However, the study is limited by its single-center design, relatively small sample size, unclear sampling framework, and insufficient discussion of the clinical significance of MIC shifts that remain within the susceptible range. Greater clarification of epidemiological context, statistical interpretation, and the implications of increasing toxin gene carriage would strengthen the manuscript.
The manuscript is well organized and clearly written. I have several minor editorial and formatting suggestions to improve clarity and consistency:
1. Please add a space after the comma and before "and" on page 2, line 51.
2. The pathogen name should be italicized on page 2, line 54, and page 10, line 234.
3. On page 4 (lines 100-101), please rephrase the sentence so that it does not begin with a number. For example: "A 500 µL aliquot of the solution was transferred to the small sample wells, and the test kit was left at room temperature for 15 minutes."
4. On page 7 (lines 191-192), please clarify that the numbers in parentheses represent the minimum and maximum ages of the patients.
5. On page 7, line 192, please remove the parentheses surrounding "72 years."
6. On page 10 (lines 230-231), please clarify what the numbers following "ST" represent.
7. Each figure legend should specify the statistical test(s) and/or analyses used to generate the data shown.
8. Gene names should be italicized on page 11 (lines 255-258).
9. The genotype should be italicized on page 14 (lines 308-309) and page 17 (line 378).
10. Please refer to Figure 4A following the sentence on page 15 (lines 313-314).
11. The sentence on page 15 (lines 315-316) should refer to Figure 4B rather than Figure 4A.
12. On page 18 (line 395), the reference lists only one author, although the cited paper has two authors. Please correct this.
13. Please add a space after the period and before "In" on page 19, line 415.

Response to Reviewers

Manuscript ID: **[Spectrum00494-26]**
Title: Longitudinal Surveillance of Antibiotic Resistance and Virulence Evolution in *C. difficile*: A Four-Year Retrospective Study of Hospitalized Patients in a Tertiary Hospital in China
Journal: Microbiology Spectrum

Professor Zhi Ruan,

We sincerely thank you and the reviewers for your constructive feedback and insightful comments on our manuscript. We carefully studied the reviewers' suggestions and made comprehensive revisions to the manuscript. We believe that these valuable suggestions will greatly enhance the rigor, clarity and overall quality of our research. Whether the manuscript is accepted or not, the reviewers' suggestions and ideas will bring significant improvements to our team and me in terms of experimental design, paper writing, etc. We would like to express our sincere gratitude again!

Below, we respond to the reviewers' comments point by point. The original reviewers' opinions are presented in bold, followed by our detailed responses. All the revisions in the revised manuscript have been marked using the "Revision" function.

Data Availability：The raw sequencing data generated in this study have been deposited in the NCBI Sequence Read Archive (SRA) database under BioProject accession number PRJNA1456209. All other associated metadata are available in the supplementary files.

Reviewer #1

General Response to Reviewer #1:
We sincerely thank Reviewer #1 for their thorough evaluation of our manuscript. We completely agree that the initial interpretation of the MIC trends was overstated. As suggested, we have carefully revised the manuscript to emphasize "reduced susceptibility" rather than "true resistance," incorporated discussions on fidaxomicin, and elaborated on the discrepancy between toxin gene carriage and protein expression. We believe these changes provide a much more balanced and scientifically rigorous interpretation of our data. Below are the individual responses and revisions to your valuable suggestions.

Comment 1:
"This study provides useful surveillance data on *C. difficile* isolates from a single hospital in China; however, the interpretation of the findings appears somewhat overstated. Although minor increases in MIC values and toxin gene prevalence were observed, the data do not yet demonstrate a true rise in antimicrobial resistance or virulence. The study also does not discuss the role of fidaxomicin, which is recommended as a first-line treatment for *C. difficile* infection, nor does it clarify that metronidazole and vancomycin are generally considered alternative or adjunct therapies. Additionally, the authors should consistently use *C. difficile*

and minimize the use of the full name to maintain clarity and adherence to standard nomenclature.
The study concludes that *C. difficile* resistance to vancomycin and metronidazole is gradually increasing, but the data do not fully support this claim. According to the results, all 114 isolates remained susceptible to both antibiotics based on EUCAST criteria, and no resistance-associated genes (such as vanA, vanB, or nim genes) were detected. Although the authors report an increase in MIC values over time, these values remain within the susceptible range, meaning there is no confirmed clinical resistance. Therefore, the evidence only indicates minor MIC variation, not true resistance development."

Response 1:
1.1 We are extremely grateful for the professional insights provided by the reviewers and fully agree. We acknowledge that since the minimum inhibitory concentration (MIC) value is still within the sensitivity range of the European Committee on Antimicrobial Susceptibility Testing (EUCAST), and no typical resistance genes (such as vanA/B, nim) have been detected, referring to this trend as "increased resistance" is inaccurate. We have now made the following revisions throughout the entire manuscript (including the title, abstract, and discussion sections) to accurately describe this phenomenon as "slow increase in MIC" or "reduced sensitivity within the sensitive range", rather than true clinical resistance:
1.1.1 Search the full text (including the title, abstract, and conclusion), and replace all instances of "increasing resistance" or "resistance increasing" with "reduced susceptibility", which is a more rigorous academic expression.
1.1.2 Address the clinical significance: In the discussion section, explicitly acknowledge that although the MIC is increasing, it has not crossed the clinical resistance breakpoint (Breakpoint), so it will not directly lead to clinical treatment failure with vancomycin or metronidazole at present. However, this indicates that we need continuous monitoring of resistance and the genome to prevent a qualitative change in the future.

1.2 Currently, Fidaxomicin has not been approved for marketing by the National Medical Products Administration (NMPA) in the Chinese mainland. Moreover, the "Guidelines for Diagnosis, Treatment and Prevention of Clostridioides difficile Infection in China (2024)" states that the first-line drugs for treating Clostridioides difficile infection in China are vancomycin and metronidazole. Therefore, this study mainly focuses on these two drugs used domestically in China. Once Fidaxomicin is approved for marketing, its efficacy in the Chinese mainland will be closely monitored. Corresponding explanations will be provided in the introduction and discussion sections.

1.3 In the entire text, except for the first occurrence of "Clostridioides difficile" in the abstract and the first occurrence in the main text, all other instances have been uniformly changed to italicized "*C. difficile*". (The correction has been made.)

Comment 2:
"The interpretation that increasing MIC values represent emerging resistance may be misleading. MIC values naturally fluctuate due to several factors, including laboratory

conditions. The authors should stick with reference method for testing the MIC for anaerobes and compare it with E-test (commercial). Because the reported MIC values remain below the resistance breakpoint, the observed trend may not represent a biologically meaningful change. How about MIC50 and MIC90".

Response 2:

2.1 We sincerely appreciate the reviewer for raising this important methodological issue. We agree that the agar dilution method is the reference standard recommended by CLSI for anaerobic bacterial susceptibility testing. However, due to the extremely high workload of the agar dilution method, in this study, the E-test was chosen instead. This is because it is operationally feasible in retrospective clinical studies, particularly suitable for clinical monitoring, and has been widely used in routine clinical microbiology laboratories. Moreover, quality control was added in each experiment to ensure the stability of the results.

E-test is a mature and widely recognized method for determining the minimum inhibitory concentration (MIC) of Clostridium difficile. This method has been widely applied in influential literature. For instance, a recent comprehensive study published in the Journal of Clinical Infectious Diseases (Dakho et al., 2022) [1] fully utilized the E-test to monitor the sensitivity of Clostridium difficile. Similarly, in a large-scale regional monitoring study conducted in South Korea, Kim et al. (2012) [2] successfully employed the E-test to provide quantitative MIC readings for multiple antimicrobials across a diverse collection of healthcare-associated C. difficile isolates. Based on these reliable precedents from the literature, we believe that our E-test results accurately reflect the phenotypic drug resistance of each isolate in our study population. To ensure that readers can clearly understand, in the revised methods section, we pointed out "Compared with the agar dilution method, there may be limitations when using the E-test. However, previous studies have shown that these two methods have a high degree of consistency in drug resistance classification. The E-test is particularly suitable for clinical monitoring due to its operability in retrospective clinical studies, and we add quality control in each experiment to ensure the stability of the experiment." We hope this explanation can address your reasonable concerns and ensure the scientific rigor of the paper.

2.2 During our discussion, we added that "We analyzed the changing trends of MIC50 and MIC90 for the two drugs over a four-year period. The results showed that the changing trends of MIC90 and the average MIC values over the four-year period were highly consistent. (See Supplementary

Table 3 Drug sensitivity results of Clostridioides difficile for vancomycin (VA) and metronidazole (MTZ) from 2021 to 2024

Year	Quantity	Vancomycin (VA) [a]MIC (μg/mL)			Metronidazole (MTZ) [b]MIC (μg/mL)		
		MIC50	MIC90	range	MIC50	MIC90	range
2021	43	0.160	0.250	0.047-0.380	0.064	0.164	0.016-0.250
2022	27	0.025	0.500	0.125-0.500	0.250	0.500	0.047-1.500

Material Table 3)"

2023	19	0.025	0.380	0.125-0.750	0.190	0.940	0.023-0.940
2024	25	0.025	0.750	0.094-0.750	0.125	0.250	0.047-2.000

a：H=33.208，P<0.05；b:H=41.990，P<0.05。

Comment 3:

"The authors also argue that *C. difficile* virulence is increasing, based on the rising proportion of strains carrying toxin genes. However, the results show a significant discrepancy between toxin gene detection (81.58%) and toxin protein expression (26.88%). This difference suggests that the presence of toxin genes does not necessarily translate into toxin production. Several biological factors could explain this discrepancy, that not explored in this MS."

Response 3:

In the discussion, add the following:" Although we found that up to 81.58% of the strains carried the toxin gene, only 26.88% actually showed positive for the toxin protein. This difference between the gene and the phenotype is reasonable. Firstly, toxin expression is strictly regulated by amino acid levels, carbon metabolism, etc. Having the gene does not mean the expression is activated. Secondly, the instructions for the Clostridium difficile glutamate dehydrogenase antigen and toxin detection kit (USA, TECHLAB) state that the detection level of toxin A in this kit is ≥ 0.63 ng/mL, the detection level of toxin B is ≥ 0.16 ng/mL, and the detection level of glutamate dehydrogenase is ≥ 0.8 ng/mL. Given the sensitivity threshold limitations of immunological detection methods, some strains with lower toxin expression levels may not be detectable."

Comment 4:

"The study utilizes WGS and resistance gene analysis, which is a strength. However, the analysis does not fully integrate genomic findings with phenotypic antibiotic susceptibility or clinical data."

Response 4:

We sincerely thank the reviewers for recognizing the value of our whole-genome sequencing method and for their insightful and forward-looking comments. We fully agree that integrating genes with phenotypic and clinical data is the best approach for infectious disease research. The main objective of this study is to depict the longitudinal and temporal dynamics of Clostridioides difficile over a four-year monitoring period. We are particularly focused on tracking the evolution trajectory of changes in antibiotic sensitivity in the pathogen reservoir and the potential for toxin production, but unfortunately, the vancomycin and metronidazole susceptibility profiles of this study were all sensitive, and no resistance genes for these two drugs were found, thus failing to establish a connection between the vancomycin and metronidazole susceptibility profiles and the genes. Regarding the study of the phenotypic and genotypic aspects of virulence, we have conducted further correlation discussions and analyses based on your suggestions. Finally, for the correlation analysis of clinical data, our team is currently conducting an independent special study

to establish a multimodal machine learning model integrating clinical phenotypes and the full-genome characteristics of the pathogen, to improve the predictive efficacy for the recurrence or critical cases of Clostridioides difficile infection. Due to the complexity of the methodology, we plan to publish the comprehensive predictive model separately. Your insightful views will further strengthen our continuous exploration in this direction and provide solid guidance for our future research.

Comment 5:
"Further research with larger sample sizes, multicenter datasets, and longer monitoring periods is needed to confirm the trends suggested and to clarify the implications for current treatment strategies. "
"The study includes 114 isolates collected over four years, averaging fewer than 30 strains per year. This relatively small sample size limits the ability to detect reliable resistance trends. Furthermore, the research was conducted in a single hospital, which restricts the generalizability of the findings. "
"The study covers a four-year period with very limited sample size, which is relatively short for assessing long-term antimicrobial resistance trends. "

Response 5:
Your suggestions are extremely valuable. We will take your valuable opinions into consideration. We will increase the sample size, recruit more centers to join, and extend the monitoring period. These will be one of the research directions that our team will continuously strive for in the future.

Response to Reviewer #3
We would like to express our sincere gratitude to Reviewer #3 for the positive evaluation of our work and the insightful comments provided. We have carefully addressed all editorial and formatting suggestions to improve the clarity and consistency of the manuscript.

General Comments:
“This study investigates longitudinal trends in antimicrobial susceptibility and virulence characteristics of Clostridioides difficile isolates collected between 2021 and 2024 at a single tertiary hospital in China. Using drug susceptibility testing, whole-genome sequencing, and bioinformatic analyses, the authors report that no fully resistant strains to vancomycin or metronidazole were identified. However, minimum inhibitory concentrations (MICs), particularly for vancomycin, increased over time, and one isolate in 2024 reached the metronidazole resistance threshold. The study also documents a rising proportion of toxin-producing strains, with all isolates in 2024 carrying toxin genes. Overall, the manuscript addresses an important clinical topic and incorporates genomic approaches to examine resistance and virulence evolution. However, the study is limited by its single-center design, relatively small sample size, unclear sampling framework, and insufficient discussion of the clinical significance of MIC shifts that remain within the susceptible range. Greater clarification of epidemiological context, statistical interpretation, and the implications of increasing toxin gene carriage would strengthen the manuscript”

Response:

We sincerely appreciate the reviewers' recognition of the clinical significance of this study. At the same time, we also acknowledge the shortcomings of the single-center design and the limited sample size. In the methods section, we have added an explanation that we determined the sampling framework through stratified sampling based on the proportion of CDI-positive cases in different wards of the entire hospital. In the revised discussion section, we further analyzed the limitations and clinical significance of the changes in MIC (within the sensitive range).

Point-by-Point Responses to Editorial/Formatting Suggestions:

1. Please add a space after the comma and before "and" on page 2, line 51.

Response 1: Corrected as suggested.

2. The pathogen name should be italicized on page 2, line 54, and page 10, line 234.

Response 2: All instances of Clostridioides difficile have been checked and italicized throughout the manuscript.

3. On page 4 (lines 100-101), please rephrase the sentence so that it does not begin with a number.

Response 3: The sentence has been rephrased to: " A 500 μL aliquot of the solution was transferred to the small sample well, and the test kit was incubated at room temperature for 15 minutes."

4. On page 7 (lines 191-192), please clarify that the numbers in parentheses represent the minimum and maximum ages of the patients.

Response 4: We have clarified this in the text: "The median age of the patients was 71 years (interquartile range *[IQR]*, 59–86 years), which was similar to the overall median age of all patients with ***C. difficile*** infection in the hospital (72 years; *IQR*, 59–83 years)"

5. On page 7, line 192, please remove the parentheses surrounding "72 years."

Response 5: Corrected as suggested..

6. On page 10 (lines 230-231), please clarify what the numbers following "ST" represent.

Response 6: We have added a definition in the revised manuscript. We clarified that "Through multi-locus sequence typing (MLST), a total of 25 distinct sequence types (STs) were identified, with each ST number representing a unique allelic profile based on seven housekeeping genes.

7. Each figure legend should specify the statistical test(s) and/or analyses used to generate the data shown.

Response 7: All figure legends have been updated to include the specific bioinformatic tools and statistical tests used . At the same time, in the methods section, the explanation of statistical analysis has been added.

8. Gene names should be italicized on page 11 (lines 255-258).

Response 8: We have italicized all gene names, including erm(B), tet(M), catP, etc., in the specified lines and throughout the document.

9. The genotype should be italicized on page 14 (lines 308-309) and page 17 (line 378).

Response 9: Corrected. All genotypes have been italicized accordingly.

10. Please refer to Figure 4A following the sentence on page 15 (lines 313-314).

Response 10: A reference to Figure 4A has been added at the end of the specified sentence to support the reported findings.

11. The sentence on page 15 (lines 315-316) should refer to Figure 4B rather than Figure 4A.

Response 11: Thank you for catching this error. The reference has been corrected to Figure 4B.

12. On page 18 (line 395), the reference lists only one author, although the cited paper has two authors. Please correct this.

Response 11: We apologize for the oversight. The citation has been corrected to include both authors ("The recurrence rate of *C. difficile* infection in this study cohort was 40.35%, consistent with the findings of Christine Leong and Sheryl Zelenitskyr").

13. Please add a space after the period and before "In" on page 19, line 415.

Response 13: Corrected as suggested.

We are grateful for the insightful comments provided by the reviewers, which have guided us in strengthening the scientific rigor and clarity of our study. We hope that the revised manuscript now meets the high standards of Microbiology Spectrum. Should there be any remaining issues, we would be pleased to address them.

We look forward to hearing from you regarding the status of our submission.

Sincerely,

[Junjie Lao#, Xianjun Wang*]

On behalf of all authors.

References

[1]Darkoh C, Keita K, Odo C, et al. Emergence of clinical clostridioides difficile isolates with decreased susceptibility to vancomycin[J]. Clinical Infectious Diseases: An Official Publication of the Infectious Diseases Society of America, 2022, 74(1): 120-126.

[2] Kim J, Kang J O, Pai H, et al. Association between PCR ribotypes and antimicrobial susceptibility among clostridium difficile isolates from healthcare-associated infections in South Korea[J]. International Journal of Antimicrobial Agents, 2012, 40(1): 24-29.

Re: Spectrum00494-26R1 (**Longitudinal Surveillance of Antibiotic Resistance and Virulence Evolution in *Clostridioides difficile*: A Four-Year Retrospective Study of Hospitalized Patients in a Tertiary Hospital in China**)

Dear Dr. Xianjun Wang,

Your manuscript has been accepted, and I am forwarding it to the ASM production staff for publication. Your paper will first be checked to make sure all elements meet the technical requirements. ASM staff will contact you if anything needs to be revised before copyediting and production can begin. Otherwise, you will be notified when your proofs are ready to be viewed.

Data Availability: ASM policy requires that data be available to the public upon online posting of the article, so please verify all links to sequence records, if present, and make sure that each number retrieves the full record of the data. If a new accession number is not linked or a link is broken, provide production staff with the correct URL for the record. If the accession numbers for new data are not publicly accessible before the expected online posting of the article, publication may be delayed; please contact ASM production staff immediately with the expected release date.

Publication Fees: For information on publication fees and which article types have charges, please visit our website. We have partnered with Copyright Clearance Center (CCC) to collect author charges. If fees apply to your paper, you will receive a message from no-reply@copyright.com with further instructions. For questions related to paying charges through RightsLink, please contact CCC at ASM_Support@copyright.com or toll free at +1-877-622-5543. CCC makes every attempt to respond to all emails within 24 hours.

ASM Membership: Corresponding authors may join or renew ASM membership to obtain discounts on publication fees. Need to upgrade your membership level? Please contact Customer Service at Service@asmusa.org.

Embargo Policy: A press release may be issued as soon as the manuscript is posted on the Spectrum Latest Articles webpage. The corresponding author will receive an email with the subject line "ASM Journals Author Services Notification" when the article is available online.

The ASM Journals program strives for constant improvement in our submission and publication process. Please tell us how we can improve your experience by taking this quick Author Survey.

Thank you for submitting your paper to Spectrum.

Sincerely,

Professor Zhi Ruan
Editor
Microbiology Spectrum
